# A rapid staining method for the detection of suberin lamellae in the root endodermis and exodermis

**DOI:** 10.5511/plantbiotechnology.25.0312a

**Published:** 2025-06-25

**Authors:** Takaki Yamauchi, Jingxia Li, Kurumi Sumi

**Affiliations:** 1 Bioscience and Biotechnology Center, Nagoya University; 2 Graduate School of Bioagricultural Sciences, Nagoya University

**Keywords:** endodermis, exodermis, fluorol yellow 088, histochemical staining, suberin lamellae

## Abstract

Histochemical detection of suberin lamellae has remarkably advanced our understanding of the roles of the apoplastic diffusion barrier in the root endodermis and the oxygen diffusion barrier in the root exodermis. Fluorol yellow 088 detects the aliphatic component of suberin and is one of the most reliable stains for detecting suberin lamellae in the endodermis and exodermis. Although fluorol yellow 088 staining can detect suberin lamellae in various plant roots by a simple procedure, conventional methods are time-consuming, as they need a long time to prepare the solution, stain, and wash the samples. Here, we propose a rapid method to minimize the time required to achieve suberin staining using root cross-sections. While conventional methods use polyethylene glycol-glycerol or lactic acid as the solvent, we found that fluorol yellow 088 readily dissolves into ethanol. This modification remarkably shortened the time required to prepare the solution, stain, and wash root cross-sections. Thus, our method will enhance the study of root anatomy and the histological development of plant roots.

Suberin comprises a glycerol-esterified polyaliphatic domain associated with an ester-bonded polyaromatic domain mainly derived from ferulic acid (Franke and Schreiber 2007; Ranathunge and Schreiber 2011). In *Arabidopsis thaliana*, suberin deposition is observed in the apoplastic compartments of the periderm, endodermis, and seed coat (Andersen et al. 2015; Franke and Schreiber 2007). In roots of rice (*Oryza sativa*) and some other species, the exodermis, a hypodermis with Casparian bands (Perumalla and Peterson 1986), deposits suberin like the endodermis (Grünhofer et al. 2023; Jiménez et al. 2024; Shiono and Matsuura 2024; Watanabe et al. 2017; Yamauchi et al. 2024). Suberized cell walls mainly function as an interface that separates adjacent tissues, and tissues from the environment (Franke and Schreiber 2007; Hose et al. 2001; Wang et al. 2025). For example, the suberized apoplastic barriers in endodermis negatively correlate with water and nutrient permeability (Grünhofer and Schreiber 2023; Ranathunge and Schreiber 2011), and the suberized exodermis associates with a barrier to radial oxygen loss and the prevention of radial water loss to the rhizosphere (Jiménez et al. 2024; Peralta Ogorek et al. 2021; Shiono et al. 2024).

The quantification of aliphatic and aromatic suberin monomers has expanded our knowledge of the chemical composition of suberin in plant roots (Kotula et al. 2017; Schreiber et al. 2005; Suresh et al. 2024). On the other hand, histochemical staining of suberin has revealed the in vivo deposition patterns of suberin lamellae in the roots (Andersen et al. 2018; Jiménez et al. 2024; Shiono et al. 2024; Wu et al. 2023). Among some suberin staining methods, fluorol yellow 088, a fluorochrome that efficiently stains lipids in fresh tissues (Brundrett et al. 1991), has been widely used to detect suberin lamellae (Andersen et al. 2018; Jiménez et al. 2024; Shiono et al. 2024). Conventional methods used polyethylene glycol-glycerol to dissolve fluorol yellow 088 (Brundrett et al. 1991). Later, lactic acid combined with saturated chloral hydrate for sample clearing was proposed as a solvent (Lux et al. 2005).

Most of the recent studies on the distribution of suberin in root cross-sections using fluorol yellow 088 stainings follow the conventional methods (Grünhofer et al. 2023; Jiménez et al. 2024; Lin et al. 2023; Manzano et al. 2024; Sexauer et al. 2021; Shiono et al. 2024; Tong et al. 2024), generally combined with the sectioning of 3–5% agarose-embedded root segments using a vibrating microtome. However, these methods are time-consuming and are unsuitable for high-throughput analysis, such as genetic screening of suberin distribution in root tissues. In the conventional method, a 0.01% (w/v) fluorol yellow 088 solution in polyethylene glycol-glycerol was prepared by heating at 90°C for 1 h (Brundrett et al. 1991). When lactic acid was used as the solvent for fluorol yellow 088, it was dissolved at 70°C for 1 h (Table 1; Lux et al. 2005). Although sample clearing is not necessarily required to detect suberin, incubation at 70°C for 1 h in lactic acid saturated with chloral hydrate is needed to remove the agarose used for sample embedding (Table 1). Subsequently, staining proceeded for 1 h at room temperature or 70°C, followed by thorough washing with distilled water (Table 1; Brundrett et al. 1991; Lux et al. 2005).

**Table 1:** Table 1. Comparison between the conventional method-based fluorol yellow 088 staining and the rapid staining method proposed in this study.

Procedure	Conventional method	Rapid method (proposed)
Preparing staining solution*	1 h	5 min
Embedding root segments	1 h	5 min
Sectioning and collecting	30 min	10 min
Clearing cross-sections	1 h	not required
Staining cross-secctions	1 h	10 min
Washing cross-sections	10 min	1 min
Total time	∼3.5 h	∼0.5 h

*The staining solution can prepare simultaneously with the other procedures.

One of the promising approaches to reduce the time and labor required is staining young root segments with fluorol yellow 088 before the sectioning of each position of the roots (Grünhofer et al. 2024). On the other hand, there is a lack of information on reducing time for the staining procedure. In the present study, we used ethanol-based fluorol yellow 088 staining combined with root cross-sectioning using a manual rotary microtome. The sectioning by vibrating microtome requires sample embedding, which takes at least 1 h for the agarose gels to melt and solidify (Table 1). Instead, using a manual rotary microtome, in which root samples are fixed between the split pith of elderberry (*Sambucus* spp.), can save time for embedding root segments and removing agarose from the cross-sections (Table 1). Fluorol yellow 088 (0.01% w/v) was easily dissolved in 99.5% ethanol at room temperature, enabling us to omit heating for 1 h to prepare the solution (Table 1). In summary, root cross-sections obtained with the manual rotary microtome were collected into 1.5 ml tubes, and the staining solution (∼200 µl) was applied to the tubes. Staining was allowed to proceed only 10 min at 60°C, followed by removal of the solution and rinsed briefly under distilled water once (Table 1). After changing to new distilled water, suberin was readily detected under a fluorescent microscope. Thus, our method effectively reduced the time for the staining procedure by at least 3 h.

To verify the accuracy of our proposed method, rice seedlings were grown under aerated conditions or stagnant conditions, which mimic flooded soils (Wiengweera et al. 1997), as previously described (Yamauchi and Nakazono 2022). After the treatment, root-cross sections at 60 mm from the root tips were prepared from the 10-mm long root segments with a manual rotary microtome. Then, the proposed rapid staining method was used to detect suberin lamellae in the root endodermis and exodermis (Figure 1). To test the effect of staining time, we incubated the staining solution at 60°C for 10 and 30 min. The results showed that 10 min was enough to detect suberin lamellae in the endodermis and exodermis in rice roots. As our method specifically detected suberin, non-suberized passage cells in the endodermis could be distinguished from the suberized endodermis (Figure 1). Moreover, the exodermal suberization, which is associated with the induction of a barrier to radial oxygen loss by stagnant conditions in rice roots (Colmer 2003; Kulichikhin et al. 2014; Shiono et al. 2014), was detected only under stagnant conditions (Figure 1), indicating that our proposed method is also applicable to evaluate the stress-induced exodermal suberization.

**Figure 1:**
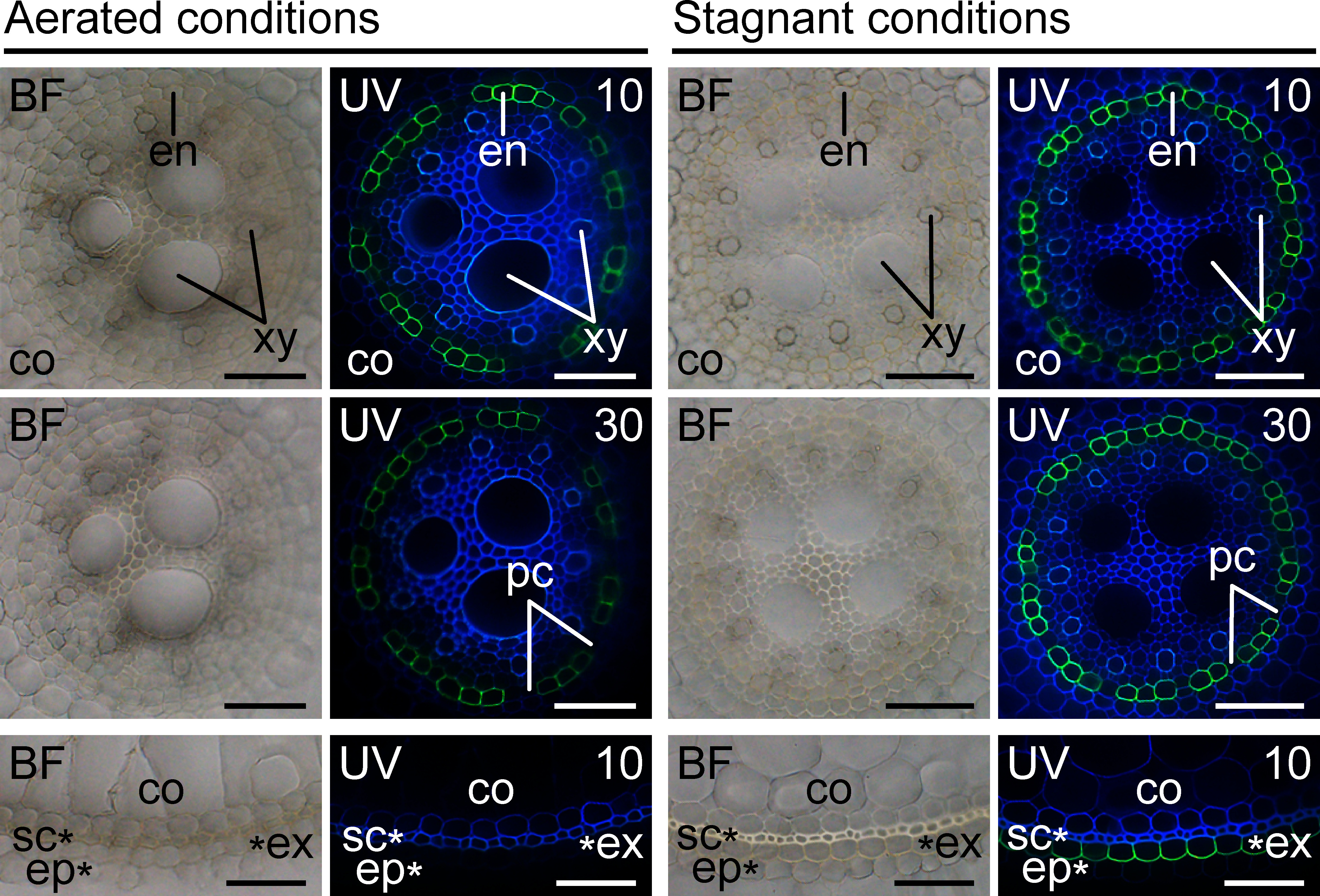
Figure 1. Suberin staining of the rice adventitious roots grown under aerated and stagnant conditions. Nine-day-old rice seedlings were further grown under aerated and stagnant conditions for seven days. Root segments were cut at 60–70 mm from the tips of adventitious roots, and cross-sections at 60 mm were prepared using a manual rotary microtome (Plant Microtome MTH-1, Nippon Medical & Chemical Instruments Co., Ltd., Japan). The aliphatic component of suberin in the cell walls of the endodermis and exodermis was stained by the ethanol-based 0.01% fluorol yellow 088 solution at 60°C for 10 min (10) or 30 min (30). Bright-field images (BF) and UV-fluorescence images (UV) are shown. Bars=50 µm. The suberin lamellae were detected with a charge-coupled device camera (DP74; Evident, Tokyo, Japan) as yellow fluorescence upon excitation by UV light (U-RFL-T; Evident) under a fluorescence microscope (BX53-FL; Evident) equipped with a U-MWU filter cube. en, endodermis; co, cortex; xy, xylem; pc, passage cells; ex, exodermis; sc, sclerenchyma; ep, epidermis.

In conclusion, the ethanol-based rapid staining method proposed herein can be useful for high-throughput analysis of suberin distribution in root tissues. To our knowledge, staining with fluorol yellow 088 has not been used for genetic screening of the patterns of suberin-lamellae deposition in plant roots. We expect that the rapid staining method will enhance our understanding of the biological significance of endodermal and exodermal suberization in plants.
